# A Quantitative Acetylomic Analysis of Early Seed Development in Rice (*Oryza sativa* L.)

**DOI:** 10.3390/ijms18071376

**Published:** 2017-06-27

**Authors:** Yifeng Wang, Yuxuan Hou, Jiehua Qiu, Zhiyong Li, Juan Zhao, Xiaohong Tong, Jian Zhang

**Affiliations:** State Key Lab of Rice Biology, China National Rice Research Institute, Hangzhou 311400, China; wangyifeng@caas.cn (Y.W.); houyuxuan@caas.cn (Y.H.); jh360@163.com (J.Q.); lzhy1418@163.com (Z.L.); zhaojuan521321@163.com (J.Z.); tongxiaohong@caas.cn (X.T.)

**Keywords:** Rice (*Oryza sativa* L.), acetylome, early seed development, post-translational modification

## Abstract

PKA (protein lysine acetylation) is a critical post-translational modification that regulates various developmental processes, including seed development. However, the acetylation events and dynamics on a proteomic scale in this process remain largely unknown, especially in rice early seed development. We report the first quantitative acetylproteomic study focused on rice early seed development by employing a mass spectral-based (MS-based), label-free approach. A total of 1817 acetylsites on 1688 acetylpeptides from 972 acetylproteins were identified in pistils and seeds at three and seven days after pollination, including 268 acetyproteins differentially acetylated among the three stages. Motif-X analysis revealed that six significantly enriched motifs, such as (DxkK), (kH) and (kY) around the acetylsites of the identified rice seed acetylproteins. Differentially acetylated proteins among the three stages, including adenosine diphosphate (ADP) -glucose pyrophosphorylases (AGPs), PDIL1-1 (protein disulfide isomerase like 1-1), hexokinases, pyruvate dehydrogenase complex (PDC) and numerous other regulators that are extensively involved in the starch and sucrose metabolism, glycolysis/gluconeogenesis, tricarboxylic acid (TCA) cycle and photosynthesis pathways during early seed development. This study greatly expanded the rice acetylome dataset, and shed novel insight into the regulatory roles of PKA in rice early seed development.

## 1. Introduction

Post-translational modification (PTM), which is a key step for functional protein maturation, refers to the covalent and generally enzymatic modification of proteins during or after protein biosynthesis. Up to now, over 300 types of experimentally verified or putative PTMs have been reported and deposited in PTM databases [1,2]. As one of the most frequent modifications, PKA (protein lysine acetylation) has been known for its critical regulatory role in various biological processes [3]. PKA was first discovered on histones, which could regulate the transcriptional level of the target genes by adjusting the status of the associated chromatin [4]. Recently, emerging evidence has shown that PKA could also occur on non-histone proteins, particularly key metabolic enzymes related to glycolysis, tricarboxylic acid (TCA) cycle in different organisms, and photosynthesis in plants. The acetylation status affects the enzymatic activities and regulates metabolic flux through these pathways [5]. Three types of proteins are required to catalyze the reversible PKA reaction. Lysine acetyltransferases (KATs) are the “writers” which functions by adding acetyl groups from acetyl-coenzyme A (acetyl-CoA) to proteins; lysine deacetylases (KDACs) work as “erasers”, which are responsible for the removal of acetyl groups from proteins; and the “readers” (acetyllysine binders) selectively interact with acetylated proteins [6]. Misfunction of some PKA-related enzymes caused severe development or growth defects in human and plants [7,8,9]. 

Rice (*Oryza sativa* L.) is one of the most important crops in the world due to its great economic and biological importance [10]. Rice seed is the carrier of the new life for progenies, but also the storage organ of nutrients which provide calories for human consumption. Rice seed development initiates from double fertilization and matures after approximately 20 days of growth. During the double fertilization, the two sperms released from pollen grain fuse with egg cell and large central cell to develop into diploid embryo and triploid endosperm respectively. In the first three DAP (Days After Pollination), embryo zygote grows from one cell to several hundred, while endosperm nuclei finish their rapid division and generate cell walls for cellularization. In this period, the maternal integuments majorly experience longitude growth and reach almost half of their maximum length. In the period 3–7 DAP, rice seeds undergo robust histodifferentiation. Apical meristem and leaf primordium differentiate out in the embryo. Meanwhile, the embryo sac starts to be filled with dividing endosperm cells, of which the outer most layers gradually differentiate into aleuronic cells, while the inner cells become starch storage cells. The maternal integument reaches its maximum length at seven DAP. Afterwards, seed development enters late stage, in which embryo becomes mature and quiescent, while endosperm cells continue to grow by cell enlargement with nutrient deposition, and finally become desiccated after 20 DAP [11,12]. 

PKA is believed to play critical roles in seed development. For example, *OsSRT1* encoding a NAD+-dependent histone deacetylase was found to regulate starch metabolism genes via reducing the histone H3K9 acetylation associated with the target genes [13], while histone acetyltransferase GCN5 (General Control Non-repressed protein 5) affected the fatty acid composition of *Arabidopsis* (*Arabidopsis thaliana*) seeds by acetylating FAD3 (Fatty Acid Desaturase 3). Therefore, profiling the acetylation sites and proteins is crucial toward understanding the regulatory roles of PKA in seed development. Owing to the recently developed antibody-based affinity enrichment methods and high sensitivity mass spectral (MS) analysis, the research community is able to investigate PKA on a proteomic scale. Thus far, at least 5000 acetylsites have been identified in human and 4000 in yeast with 2850 and 1050 acetylproteins, respectively [3]. In plants, over 5000 acetylsites on 2900 acetylproteins have been profiled in various species, including *Arabidopsis*, *rice*, *wheat*, *grape*, *potato* and *strawberry*, which greatly enhanced our understanding of the regulatory roles of PKA in plants [14,15,16,17,18,19,20,21,22,23,24]. However, all the works mentioned above were done in a qualitative manner, which only provided a static landscape of this dynamic modification. Here, we reported the first quantitative acetylomic case in plants with a special focus on the rice early seed development. The results obtained from this work aimed to provide an overall view of the acetylation events as well as the regulatory roles of PKA during early seed development in rice. 

## 2. Results

### 2.1. Global Profiling of PKA (Protein Lysine Acetylation) in Rice Pistil and Developing Seeds

**Figure 1:**
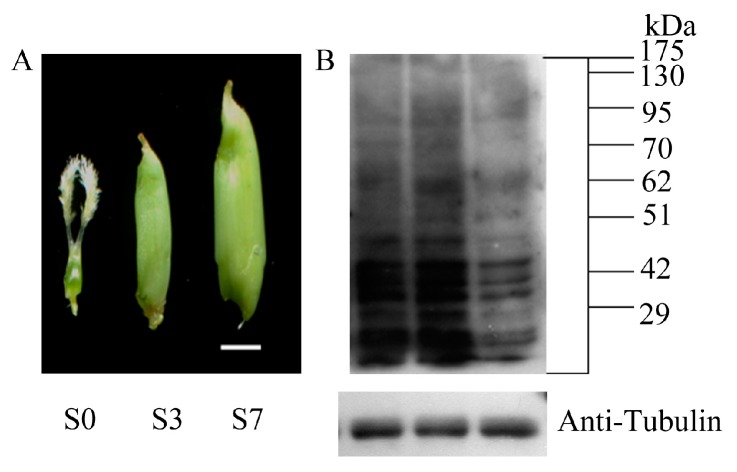
(**A**) Seed morphology of the pistil (S0), seed at three DAP (Day after pollination) (S3) and seven DAP (S7) from left to right. Scale bar = 2 mm; and (**B**) Western blot analysis of the acetylation dynamics in early rice seed development by using anti-acetyl lysine antibodies. Equal amount of proteins (20 µg) were used and the sample order from left to right: S0, S3 and S7. Anti-tubulin was used as an internal control for normalization. Molecular mass markers were shown (kDa).

**Figure 2:**
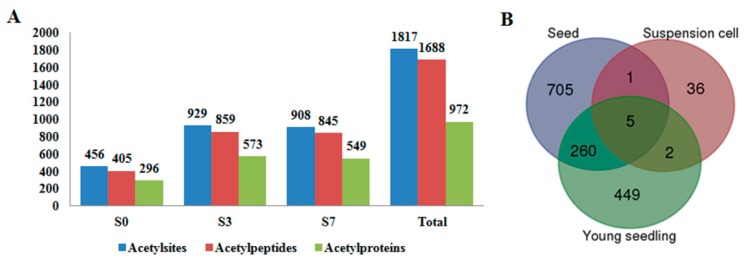
(**A**) The counts of acetylsites, acetylpeptides and acetylproteins in S0, S3 and S7, respectively; and (**B**) Venn diagram showing the overlap between our identified acetylproteins and the published acetylomes data in rice suspension cells and young seedlings [20,23], respectively.

To dissect the regulatory roles of PKA in the rice early seed development, we performed a quantitative, MS-based acetylproteomic study for the seeds at three typical developing stages, namely unpollinated pistil (S0), seed at three DAP (Day After Pollination) (S3) and seven DAP (S7) of Nipponbare (*Oryza sativa* L. ssp japonica) with three biological replicates (Figure 1A). The three time points represent the pre-pollination, post-pollination, fast proliferation and histodifferentiation status of rice seed. Firstly, we analyzed the three protein samples by Western blot against a pan anti-acetylation antibody. As shown in Figure 1B, the acetylation patterns of the total seed proteins were altered along with the seed development from S0 to S7, indicating that PKA may play important roles in early rice seed development. Intriguingly, multiple non-histone bands (>25 KD) were also detected to be acetylated, which was consistent with the previous reports [16,20] (Figure 1B). The acetylomic MS identification collectively profiled 456, 929 and 908 acetylsites on 405, 859 and 845 acetylpeptides, which corresponds to 296, 573 and 549 acetylproteins from S0, S3 and S7, respectively. After removing the redundancies, totally 1817 acetylsites were obtained on 1688 acetylpeptides from 972 acetylproteins in this study, which represented the largest acetylome dataset in plants thus far (Figure 2A; Table 1; Table S1). Among the 972 acetylproteins, 97.5% (948) were non-histone proteins, which was comparable to the non-histone acetyprotein ratio in rice suspension cell (86.3%) and young seedling (97.2%) [20,23]. Transcription factor (TF) is a major category of genes in rice genome. However, we only detected six acetylated TFs in the 972 seed acetylproteins. Given that 2408 of the 39,045 rice non-TE (Transposable element) genes are TFs in the rice genome, the results suggested that PKA preferentially occur in non-TF proteins in rice seeds [25,26]. By comparing with the previously identified acetylproteins in rice [20,23], we found that one and 260 of our acetylproteins were overlapped with the acetylome data in rice suspension cells and young seedlings, respectively, while only five acetylproteins exists in all the three datasets. Thus, the current study contributed 705 novel acetylproteins to the rice acetylome (Figure 2B).

### 2.2. Conserved Motifs Flanking the Acetylsites

**Figure 3:**
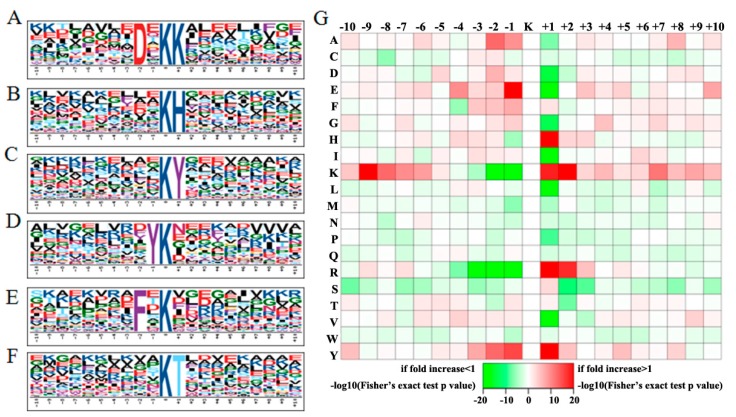
(**A**–**F**) Motif-X analysis of over-represented motifs around the acetylsites of the identified rice seed acetylproteins: **A**, (DxkK); **B**, (Kh); **C**, (kY); **D**, (Yk); **E**, (Fxk); **F**, (kT); and (**G**) Icelogo heat map of the 21 amino acid compositions of the acetylated site showing the frequency of the different amino acids in specific positions flanking the acetylated lysine. The different colors of blocks in (**G**) represent the preference of each residue in the position of a 21 amino acid-long sequence context.

To search for the acetylation conserved motifs in rice, the context amino acid sequence around the acetylsites were extracted for Motif-X analysis (Available online: http://motif-x.med.harvard.edu/) [27]. Figure 3A–F depicted the top 6 over-represented motifs in this study (Fold increase > 2.5, *p* < 0.01). (DxkK) (x for any residues, k for acetylated lysine) and (kH) are the most enriched motifs with over six-fold increases when compared with the background matches, suggesting that positive charged (basic) residues like K and H are favored by PKA. Followed enriched motifs are (kY) and (Yk) containing a Y in the close upstream or downstream of the acetylsites. We also found that over 100 acetylsites were located within motifs (Fxk) and (kT). By constructing Icelogo heat maps, we assessed the preference of each residue in the position of a 21 amino acid-long sequence context (Figure 3G). Biased residue distributions were concentrated in the region from −3 to +2 when the acetylated lysine is considered as 0, which indicated this region may be important for the catalyzation by PKA enzymes. In the +1 position, residues H, K, R and Y were more highly enriched than other residues, whereas residues E, G, I, L and V were less preferentially presented. Meanwhile, residues K and R were also over-represented in position +2. On the downstream of the acetylsites, residues E and Y were frequently detected, while K and R were barely found from −3 to −1. These motif model and residue preferences could provide valuable information for the acetylsites prediction of the unknown acetylproteins.

### 2.3. Differentially Acetylated (DA) Proteins in Rice Developing Seeds

**Figure 4:**
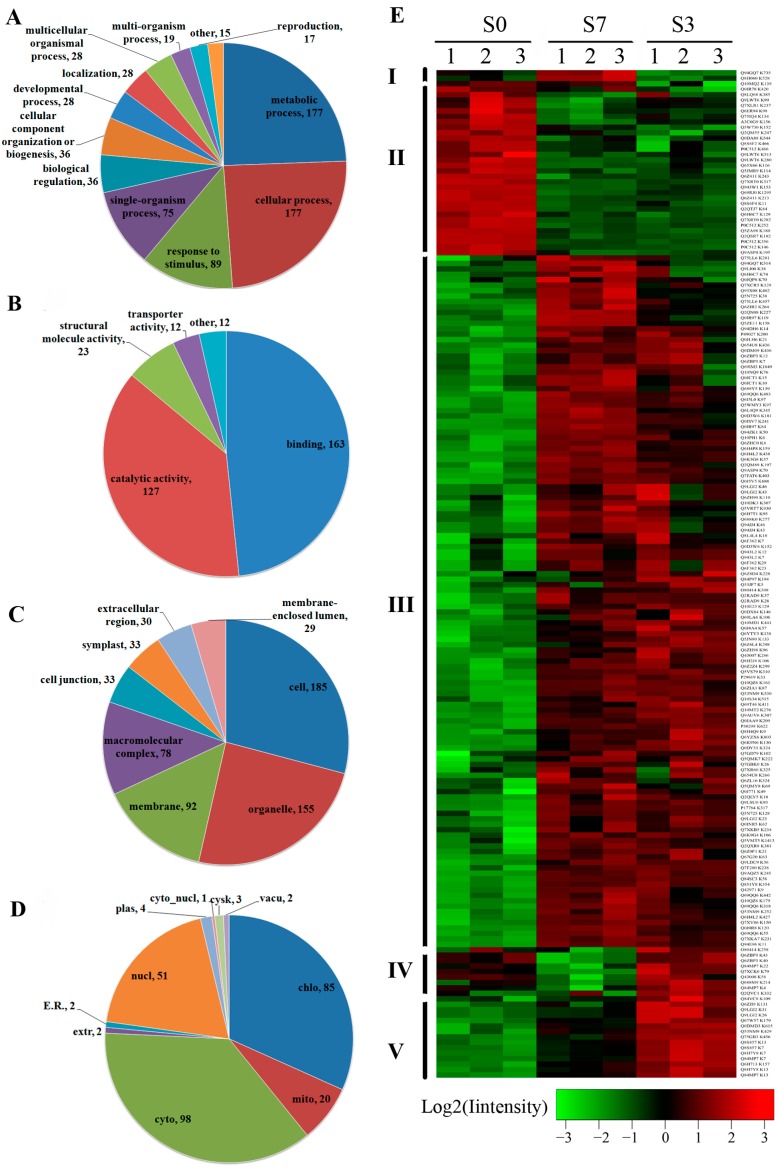
GO analysis of differentially acetylated proteins in terms of: biological process (**A**); molecular function (**B**); cellular component (**C**); and subcellular location (**D**), and (**E**) acetylation quantitation heat map of differentially acetylated (DA) proteins in S0, S7 and S3. The sample order from left to right: S0, S7 and S3. Color bar at the bottom represents the log2 acetylation site quantitation values. Green, black and red indicate the low, medium and high acetylation intensity, respectively. The abbreviations in (**D**) represent as follows: E.R.: endoplasmic reticulum; extr: extracellular matrix; cyto: cytoplasm; mito: mitochondrial; chlo: chloroplast; vacu: vacuole; cysk: cytoplasmic skeleton; cyto_nucl: cytoplasm nuclear; plas: plasma membrane; nucl: nuclear.

Employing a label-free approach, we quantified the acetylation intensity of each peptide and finally identified 370 differentially acetylated peptides on 268 acetyproteins (|fold change| > 1.5, *p* < 0.05) (Table S2). During the transition from S0 to S3, 160 and 62 proteins were up-acetylated and down-acetylated, respectively, which may be a consequence of the double fertilization. In S7/S3, 35 and 85 proteins were up-acetylated and down-acetylated, respectively, which indicated these proteins may be involved in the robust histodifferentiation and proliferation during early seed development. Meanwhile, 191 and 59 proteins were up- and down-acetylated, respectively, in S7/S0. A Gene ontology (GO) functional characterization revealed that the majority of the DA proteins were involved in “metabolic process”, “cellular process” and “response to stimulus” under the category of “biological process” (Figure 4A). In support of the “metabolic process” annotation, the “molecular function” of the DA proteins was mostly categorized to “catalytic activity” and “binding” (Figure 4B). In terms of “cellular component”, 185 of the DA proteins were located in the cell, 155 in “organelle”, which totally occupy a ratio of 67.3% of the protein analyzed (Figure 4C). We further investigated the distribution of DA proteins in various subcellular compartments. The results indicated that the majority of acetylproteins were located in cytoplasm (98, 36.6%) and chloroplast (85, 31.7%). A number of DA proteins also distributed in nucleus (51, 19.0%) and mitochondrial (20, 7.5%), while only a small part of the proteins were predicted to be in other compartments like extracellular, vacuole and Endoplasmic reticulum (ER) (Figure 4D). 

Based on the acetylation intensities in the three stages of seed development, a hierarchical clustering analysis was conducted to visualize the acetylation dynamics of each DA protein (Figure 4E). The 268 DA proteins were primarily divided into five clusters. Proteins in cluster II had the highest acetylation intensity in S0, while relatively lower intensity in S3 and S7 stages, which indicated that the deacetylation occurred on these DA proteins after double fertilization. Notably, proteins in cluster IV had higher acetylation level in S0 and S3 when compared with S7. On the other hand, double fertilization seemed to up-regulate the acetylation level of cluster III, IV and V proteins. During the transition from S3 to S7 (a period in which developing seeds undergo fast differentiation and proliferation), the acetylation was increased on cluster I and III proteins, but decreased on cluster IV and V proteins. The diverse acetylation pattern hinted the different roles of these proteins in early seed development. Meanwhile, the co-acetylation pattern of the proteins in the same clusters also indicated their roles in the similar pathways. 

### 2.4. Functional Domain and Kyoto Encyclopedia of Genes and Genomes (KEGG) Pathway Enrichment Analysis of the DA Proteins

**Figure 5:**
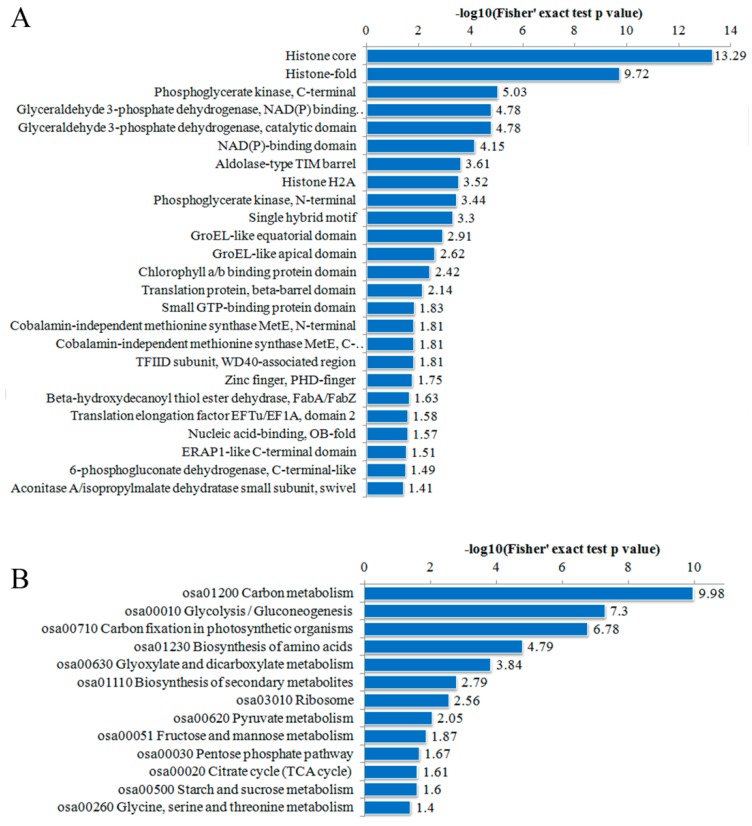
Enrichment analysis of DA proteins based on: protein domain (**A**); and kyoto encyclopedia of genes and genomes (KEGG) pathway (**B**).

To better understand the potential functions of the DA proteins, we conducted a protein domain enrichment assay by searching against the interpro database (Available online: http://www.ebi.ac.uk/interpro/) [28]. A total of 25 categories of protein domains were enriched in these DA proteins (*p* < 0.01) (Figure 5A). As expected, the top 2 enriched protein domains were histone-related, which is consistent with the previous report in rice seedlings [23]. The followed most enriched protein domain was “phoshoglycerate kinase”, a major enzyme functioning in glycolysis in the first adenosine triphosphate (ATP) -generating step of the glycolytic pathway. Moreover, the enrichment of some other glycolysis-related protein domains such as “glyceraldehyde-3-phosphate dehydrogenase”, “NAD(P) (pyridine nucleotide)-binding” and “Aldolase-type TIM barrel (a conserved protein fold consisting of eight α-helices and eight parallel β-strands that alternate along the peptide backbone)” were also detected, which indicated the extensive involvement of PKA in carbohydrate metabolic pathways during early seed development. 

We also performed KEGG analysis to assess the potential pathways in which DA protein may be involved [23]. The results showed that metabolic-related pathways, including carbon metabolism, glycolysis, and carbon fixation in photosynthesis, were predominantly over-represented (*p* < 0.01). The DA proteins also participated in other processes such as biosynthesis of amino acids, biosynthesis of secondary metabolites and ribosome (Figure 5B).

### 2.5. Protein–Protein Interaction (PPI) Analysis of DA Proteins

**Figure 6:**
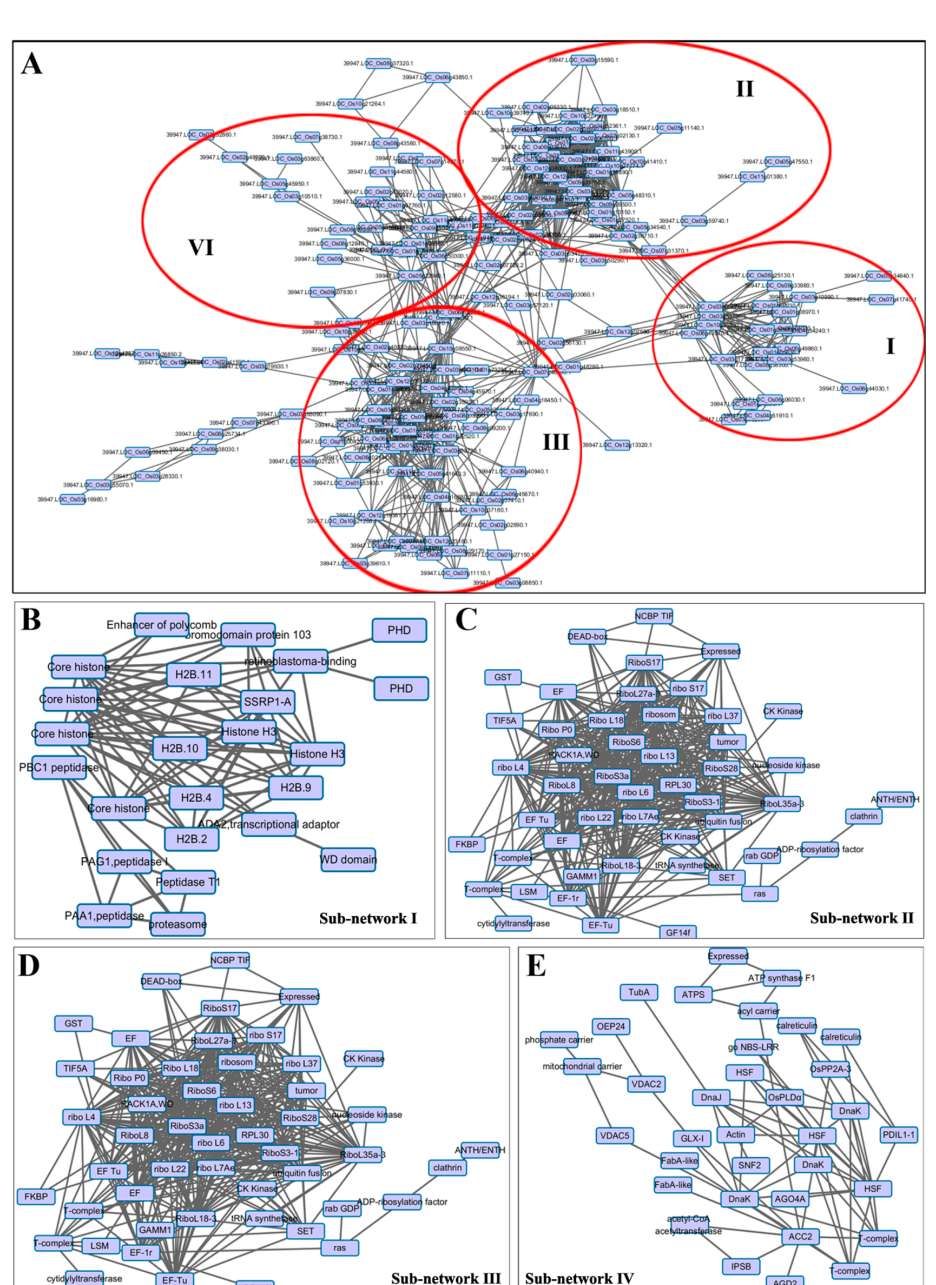
Protein–protein interaction (PPI) network of DA proteins identified in this study. (**A**) the whole PPI network of all DA proteins; the red circles represent the sub-network groups of DA proteins; (**B**) sub-network I of DA proteins associated with histone proteins; (**C**) sub-network II of DA proteins associated with ribosomal proteins; (**D**) sub-network III of DA proteins involved in glycometabolism and photosynthesis; and (**E**) sub-network IV of DA proteins associated with the enzymes catalyzing PKA.

To understand how are the DA proteins functionally related with each other, a protein–protein interaction (PPI) network was constructed by using the String 10.0 (Search Tool for the Retrieval of Interacting Genes/Proteins) (Available online: http://string-db.org/) [29] and visualized by Cytoscape [30]. The results contained 186 nodes (proteins) and 986 edges (interaction-ship), indicating a highly profound network of the acetylproteins in seed development (Figure 6A; Table S3). We tentatively divided the whole network into four sub-networks for an in-depth insight of their interactions. The sub-network I was majorly comprised of various histone proteins, including H2Bs and H3s (Figure 6B). As a key type of epigenetic regulation, the differential acetylation of these histone proteins may impact the transcriptional level of the associated genes, and thus coordinate the seed development process. More interestingly, we found over 20 ribosomal proteins in sub-network II (Figure 6C). Given that the ribosome is the location for protein “translation”, differential acetylation on ribosome machinery may be another way to control the quantity of seed development-related proteins. Sub-network III harbored a large number of phosphoglycine kinases and dehydrogenases, which were functionally related to glycometabolism. Meanwhile, the identification of three chlorophyll binding proteins and one photosystem I protein indicated the important roles of PKA in photosynthesis (Figure 6D). Sub-network IV included an interaction map of acetyl-CoA transferase-, acetyl-CoA carboylase-acetyl carrier, and hence the enzymes catalyzing PKA could also be subjected to the regulation of protein acetylations (Figure 6E). 

## 3. Discussion

Recently, emerging evidence has shown that PKA on either histone proteins or non-histone proteins play essential regulatory roles in various biological processes. Toward understanding the PKA functions in plants, acetylome analyses have been performed in a few species and over 5000 acetylsites on around 3000 acetyproteins have been identified, which largely expand our knowledge in this field. Nevertheless, the identified acetysites were just a tip of the iceberg, considering the high frequency of PKA in plant proteome [3]. Moreover, the previous plant acetylome analyses were mostly qualitative descriptions of PKA in a certain tissue or growth stage, which only provided a static insight of this highly dynamic PTM type. Here, we report the first quantitative profiling of PKA dynamics during early seed development in rice. A total of 1817 acetylsites on 1688 acetylpeptides from 972 acetylproteins, including 705 novel acetylproteins, were identified. In total, 268 acetyproteins were differentially acetylated (|fold change| > 1.5, *p* < 0.05) among the S0, S3 and S7 stages, indicating the key roles of PKA on these proteins during early seed development. 

As expected, several of the DA proteins were reported to be important seed development regulators. ADP-glucose pyrophosphorylases (AGPs) are step-limiting enzymes for the synthesis of starch, a major reserve of rice endosperm [31]. In rice, the AGP family contains two small subunit components and four large subunit components which form a heterotetrameric complex to catalyze the production of ADP-Glc for α-1,4-glucan synthesis. Previous studies demonstrated that mutation of *OsAGPL2* and *OsAGPS2* caused shrunken endosperms due to the remarkable reduction in starch synthesis [32]. In our acetylome data, we found that the acetylation on OsAGPS2 was unchanged between S0 and S3, but highly mounted at S7 (Table S2). Given that starch biosynthesis is in “off” before 5 DAP and starts after that, the accumulated acetylation detected on S7 suggested a positive role of the acetylation on OsAGPS2 activity. PDIL1-1 (protein disulfide isomerase like 1-1) is known for its function in the maturation of storage protein glutelins. During seed development, the precursors of storage protein glutelins are initially synthesized on the endoplasmic reticulum (ER), and then exported to protein storage vacuole, where the precursor subunits are interlinked by disulfide chain for protein maturation [33]. PDIL1-1 is largely restricted to the cisternal ER, and may catalyze the formation and breakage of disulfide bonds within glutelins to facilitate their maturation. PDIL1-1 only works under the condition that glutelin synthesis rate exceeds the export rate. Accumulation of glutelin in ER initiates correct folding of the protein, conferring on it a conformation that is competent for export from the ER. In accordance, *pdil1-1* mutants exhibited elevated accumulation of proglutelin [34,35]. Intriguingly, the current study revealed an up-acetylation pattern of PDIL1-1 during seed development, and the plateau intensity reached at S7, a stage that PDIL1-1 was believed to be highly activated for robust storage protein synthesis (Table S2),which indicated that PKA may be a switch controlling the activity of PDIL1-1.

During early seed development, sucrose metabolism is a major source providing energy and carbon skeleton for the vigorous histodifferentiation. The transported sugars in the sink organ are predominant hydrolyzed into glucose and fructose, which would substantially increase the hexose content. Subsequently, hexoses are incorporated into the central metabolism like glycolysis and TCA cycles to elevate the energy level [36]. In support to the previous findings, we found that the starch and sucrose metabolic pathway was over-represented in the acetylome (Figure S1). In this pathway, eleven enzymes like glucose-1-phosphate adenylyltransferase, UDP (Uridine diphosphate) -glucose 6-dehydrogenase, sucrose synthase I and fructose kinase were found to be differentially acetylated (Figure S1; Table S2). The acetylation levels of these enzymes were positively correlated with the histodifferentiation status in seeds, suggesting the important regulatory roles of acetylation in sucrose metabolism.

The energy for seed development is supplied by the central metabolic pathways, including glycolysis/gluconeogenesis and tricarboxylic acid (TCA) cycle. Previous seed proteomic studies identified a large number of glycolytic enzymes with significant abundance change, though the enzyme numbers and accumulation patterns varied from species, which indicated the extensive involvement of glycolysis in this process [37]. Glycolysis catalyzes the conversion of glucose into pyruvate and generation of small amount of ATP (energy) and NADH (reducing power), while gluconeogenesis synthesizes glucose from noncarbohydrate precursors. Our KEGG pathway assay of the DA proteins revealed the significant enrichment of glycolysis/gluconeogenesis pathway with 18 DA enzymes identified, including hexokinase, enolase, fructose-bisphosphate aldolase, phosphoglycerate kinase, glyceraldehyde-3-phosphate dehydrogenase and pyruvate decarboxylase, which participate in various key steps of the glycolytic pathway (*p* < 0.01, Table S2; Figure S2). For example, hexokinases are the enzymes catalyzing the phosphorylation of glucose into glucose 6-phsphate (G6P), which is the first step of the preparatory phase of glycolysis [38]. Meanwhile, pyruvate kinases regulate the last step to produce ATP and pyruvate. Moreover, most of these enzymes were up-acetylated during seed development, which indicated a positive role of acetylation in this pathway (Table S2). 

The conversion of pyruvate into acetyl-CoA, by the pyruvate dehydrogenase complex (PDC) link the glycolytic pathway to the TCA cycle which is another key metabolic pathway that unifies carbohydrate, fat, and protein metabolism [39]. It has been clear that PDC acts as a switch for the transition of carbon flux through from the energy production to the deposition of storage reserve, because PDC declines was observed to coincide with the deposition of storage reserves [37]. As expected, a PDC component (Q2QM55) was down-acetylated during the early development of seeds (Table S2), suggesting that the PDC activity could be controlled by acetylation modification. Due to the decreased flux through the TCA cycle, less production of NADH reduced the consumption of oxygen, which would help seeds to overcome the shortage of internal oxygen and relieve damage to cells in development [40]. In this study, we found that these DA proteins covered 11 TCA-related enzymes such as malate dehydrogenase, isocitrate dehydrogenase, succinyl-CoA ligase and aconitate hydratase, which made the TCA cycle an enriched pathway (Table S2; Figure S3). It was interesting that the acetylation intensity of these TCA-related proteins were up-acetylated in S3/S0 (*p* < 0.05), but remained unchanged in S7/S3 (*p* > 0.05) (Table S2). The up-regulated TCA pathway from S0 to S3 may be required to provide the energy for hitodefferentiation. However, though we did not observe the down-acetylation of the TCA-related enzymes enzymes from S3 to S7, the unchanged acetylation intensity could be an indicator of the down-acetylation of TCA enzymes after S7, when storage reserve deposition just started and TCA became inactivated. To confirm this hypothesis, more acetylomic analyses of stages after S7 need to be further investigated.

Rice and wheat seeds have highly accumulated photosynthesis-related proteins, particularly at the stages of histodifferentiation and early stage of reserve deposition, while it decreased with the maturation of seeds [41,42]. Photosynthesis in seeds was believed to support starch synthesis and supply oxygen to the growing lateral and peripheral regions of the endosperm [41,43]. The current study demonstrated an extensive participation of PKA in the regulation of photosynthesis in seeds, as 10 DA proteins were found to be functionally relevant to photosynthesis (Figure S4). These DA proteins were highly enriched in the pathway of “photosynthesis–antenna proteins” and “Carbon fixation”. The antenna complex is an array of proteins and chlorophyll molecules responsible for the energy capture from lights [44]. As revealed by our protein–protein interaction analysis, three DA antenna proteins (Q6Z411, Q5ZA98 and Q10HD0) may work as a complex in this process (Figure 6D; Table S2). The acetylated carbon fixation enzymes included glyceraldehyde-3-phosphate dehydrogenase, fructose-bisphosphate aldolase, phosphoglycerate kinase and malate dehydrogenase. Notably, we identified the acetylation on both the large chain and small chain subunits of the Ribulose bisphosphate carboxylase (RuBisCO), which is one of the rate-limiting enzymes for carbon fixations [45]. The large chain and small chain subunits were identified in the mass spectral (MS) seven and two times, respectively, suggesting the acetylation status was highly reliable. Moreover, we also noticed that the RuBisCOs were down-acetylated at S7, which was in agreement with the previous report that photosynthesis activity gradually decreases with the seed development [41,42]. 

## 4. Materials and Methods 

### 4.1. Collection and Preparation of Plant Materials

The collection and preparation of pistil, three DAP and seven DAP seeds of Nipponbare were performed as described previously with three biological replicates [12]. Briefly, the mature pistils were manually dissected and collected before the panicles fully headed out. For the developing seeds, each spikelet was labeled on the day of anthesis, and seeds were manually collected with glumes removed at three DAP (around 6 mm in length) and seven DAP (around 10 mm in length).

### 4.2. Protein Extraction

Sample fine powder was transferred to a 5 mL centrifuge tube containing lysis buffer (8 M urea, 2 mM EDTA, 3 μM TSA, 50 mM NAM, 10 mM DTT (DL-Dithiothreitol) and 1% Roche Protease Inhibitor (Scientz, Ningbo, China). After removing the debris by centrifugation, the protein in the supernatant was precipitated with ice-cold 15% TCA, and washed with cold acetone for three times. The protein was redissolved in buffer (8 M urea, 100 mM NH4CO3, pH 8.0), and then quantified with a 2-D Quant kit (GE Healthcare, Chicago, IL, USA) according to the manufacturer’s instructions.

### 4.3. Western Blotting

Around 20 μg of each sample was separated by 10% SDS-PAGE gels and transferred to a polyvinylidine fluoride fluoropolymer (PVDF) membrane (0.45 μm, Millipore, Darmstadt, Germany) using Trans-Blot Turbo transfer system (Bio-Rad, California, CA, USA). The membrane was blocked with TBST (10 mM Tris-HCl, 150 mM NaCl, and 0.05% Tween 20, pH 8.0) containing 5% BSA at 4 °C overnight. The target protein bands were sequentially detected by acetyl lysine primary antibodies (1:1000 dilution in TBST) (ImmuneChem, Burnaby, BC, Canada) and HRP (horseradish peroxidase–conjugated) secondary antibodies (1:1000 dilution in TBST) (Beyotime Company, Shanghai, China), and finally visualized with enhanced chemiluminescence reagent (Thermo, Waltham, MA, USA).

### 4.4. Protein Digestion and Acetylpeptide Enrichment

The protein digestion and acetylpeptide enrichment were performed as follows. Firstly, the proteins were sequentially reduced with 10 mM DTT for 1 h at 37 °C, then alkylated with 20 mM IAA for 45 min at room temperature in darkness and diluted by adding 100 mM NH_4_CO_3_ to urea concentration lesser than 2 M, and digested with trypsin at 1:50 trypsin: protein mass ratio overnight for the first time and at 1:100 trypsin: protein mass ratio 4 h for the second time. After this, treated peptides were dissolved in NETN buffer (100 mM NaCl, 1 mM EDTA, 50 mM Tris-HCl, 0.5% NP-40, pH 8.0), the tryptic peptides were incubated with pre-washed antibody beads (PTM Biolabs, Hangzhou, China) by gentle shaking at 4 °C overnight to enrich Kac peptides. Then the bound peptides were washed four times with NETN buffer and twice with ddH_2_O, eluted from the beads with 0.1% TFA. The eluates were subsequently vacuum dried using a SpeedVac and cleaned with C18 ZipTips (Millipore, Darmstadt, Germany) according to the manufacturer’s instructions for LC-MS/MS analysis. 

### 4.5. Liquid Chromatography Tandem Mass Spectrometry (LC-MS/MS) Analysis and Database Search

The LC-MS/MS analysis and database search were performed as follows. Firstly, peptides were sequentially dissolved in 0.1% FA (formic acid), directly loaded onto a reversed-phase pre-column (Thermo, Waltham, MA, USA) and separated with a reversed-phase analytical column (Thermo, Waltham, MA, USA.). The gradient solvent B, (0.1% FA in 98% ACN) was increased from 6% to 22% for 24 min, 22% to 40% for 8 min and 80% in 5 min, and then held at 80% for the last 3 min with a constant flow rate of 300 µL∙min^−1^ on an Easy-nLC1000 UPLC system (Thermo, Waltham, MA, USA) used to separate the enriched acetylpeptides. Then, full-scan mass spectra identification was analyzed by Q Exactive ^TM^ mass spectrometer (Thermo, Waltham, MA, USA) (mass range: 350–1800 mz^−1^; resolution: 7000). The generated raw data was used to search against uniprot_*Oryza sativa* database by using MaxQuant search engine (v.1.4.1.2) [46]. The parameters in MaxQuant were as follows: Trypsin/P was specified as cleavage enzyme allowing up to 4 missing cleavages, 5 modifications per peptide and 5 charges; mass error was set to 10 ppm for precursor ions and 0.02 Da for fragment ions; carbamidomethylation on Cys was specified as fixed modification and oxidation on Met, acetylation on Lys protein N-terminal were specified as variable modifications; minimum peptide length was set at 7; FDR (False discovery rates) thresholds for protein, peptide and modification site was adjusted to <1% and minimum score for modified peptides was set >40, while the other parameters were set to default values.

## 5. Conclusions

In this study, the first quantitative acetylproteomic analysis focus on rice early seed development was performed by employing a MS-based, label-free approach. A total of 1817 acetylsites on 1688 phosphopeptides from 972 acetylproteins were identified in pistils and seeds at three and seven DAP (Days After Pollination). In total, 268 acetyproteins were differentially acetylated among the three stages, including several rice seed development related proteins, which indicated that protein lysine acetylation (PKA) could play an important role in the regulatory mechanism of rice early seed development. In other words, the data obtained from this study not only enhanced our understanding of the rice acetylproteome dataset, but also shed novel insight into the roles of PKA in rice seed development. Data Availability: The mass spectrometry proteomics data have been deposited to the ProteomeXchange Consortium [47] via the PRIDE (The PRIDE PRoteomics IDEntifications) partner repository with the dataset identifier PXD005982.

## Figures and Tables

**Table 1:** Comparison of the reported plant acetylomes with the current study.

Species	Tissue	Acetylsites	Acetylproteins	Reference
*Triticum aestivum*	leaf	416	277	[14]
*Oryza sativa*	Suspension cell, young seedling	1403	760	[20,23]
*Arabidopsis thaliana*	Suspension cell, young seedling	398	251	[17,18]
*Glycine max*	developing seeds	190	121	[22]
*Pisum sativum*	seedling	664	358	[21]
*Solanum tuberosum*	tuber	35	31	[24]
*Fragaria X ananassa*	leaf	1392	684	[16]
*Vitis vinifera*	mesocarp and exocarp	138	97	[19]
*Brachypodium distachyon*	leaf	636	353	[15]
*Oryza sativa*	pistil and developing seeds	1817	972	this study

## Supplementary Materials

Supplementary materials can be found at www.mdpi.com/1422-0067/18/7/1376/s1.
