# Supplementary material for: A Quantitative Acetylomic Analysis of Early Seed Development in Rice (Oryza sativa L.)
Source: Int J Mol Sci. 2017 Jun 27;18(7):1376. doi: 10.3390/ijms18071376 (PMC5535869; doi:10.3390/ijms18071376)
Supplement: Supplementary file 1 [file ijms-18-01376-s001.zip › supplementary/ijms-199105 supplementary Figures .docx]

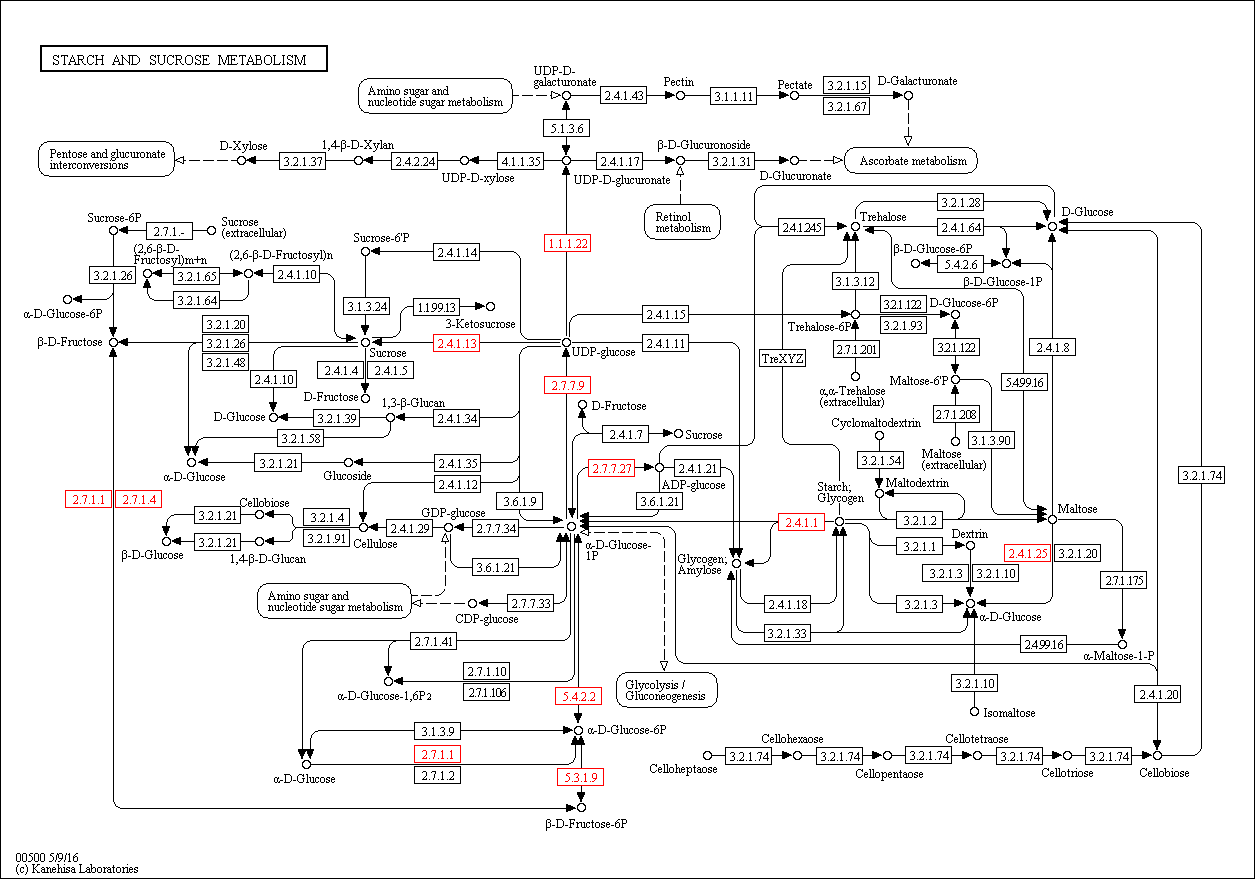


**Figure S1.** Enriched KEGG pathways of DA proteins involved in starch and sucrose metabolism. The identified DA proteins in this study were marked in red.


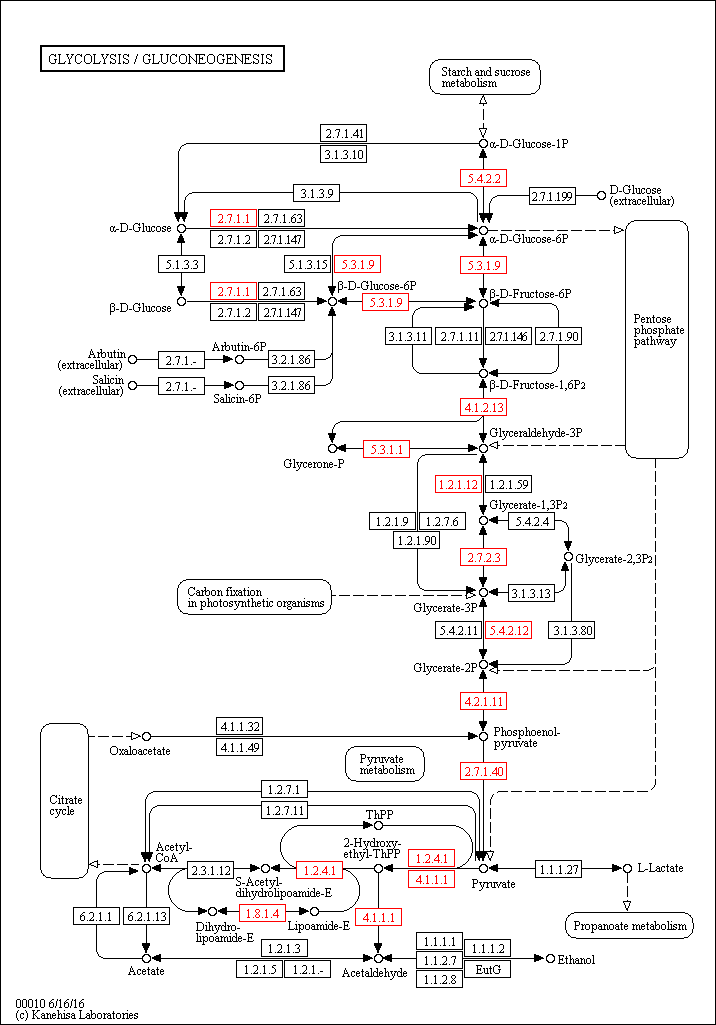


**Figure S2.** Enriched KEGG pathways of DA proteins involved in glycolysis/glyconeogenesis. The identified DA proteins in this study were marked in red.


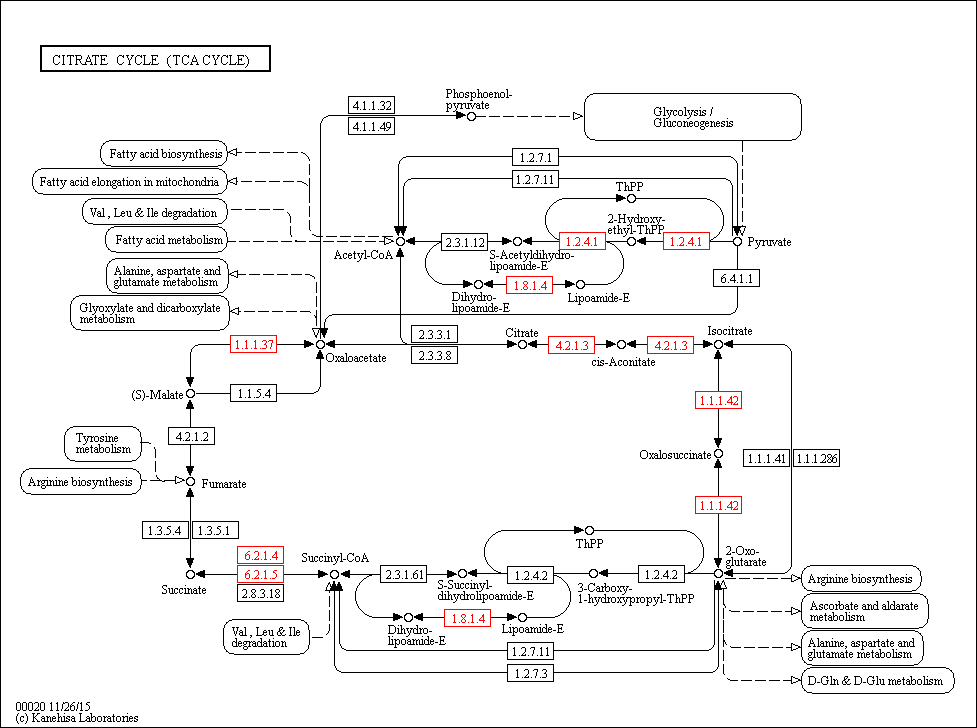


**Figure S3.** Enriched KEGG pathways of DA proteins involved in citrate cycle (TCA cycle). The identified DA proteins in this study were marked in red.


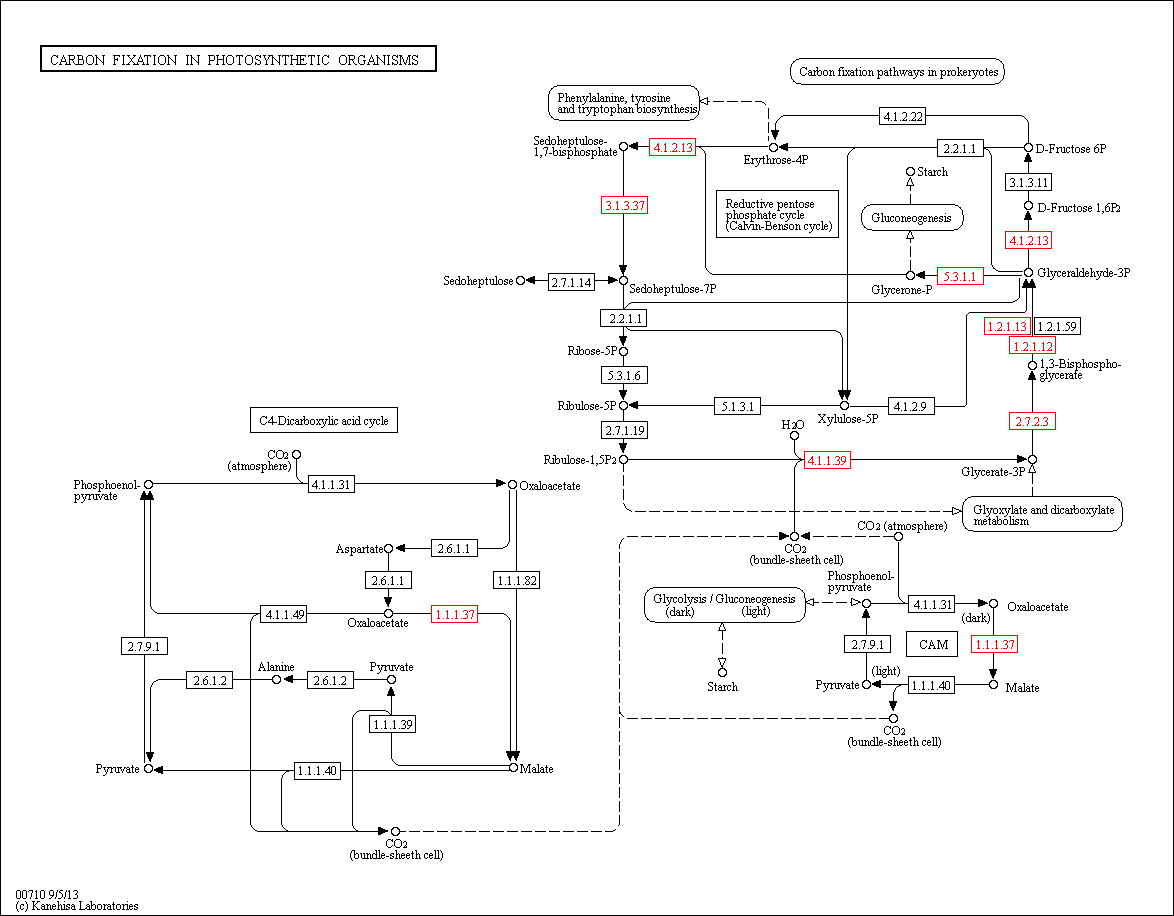


**Figure S4.** Enriched KEGG pathways of DA proteins involved in carbon fixation in photosynthetic organisms. The identified DA proteins in this study were marked in red.
